# *Moringa oleifera* leaves protein suppresses T-lymphoblastic leukemogenesis via MAPK/AKT signaling modulation of apoptotic activation and autophagic flux regulation

**DOI:** 10.3389/fimmu.2025.1546189

**Published:** 2025-04-01

**Authors:** Xiaoxue Liu, Hao Yang, Shanna Xie, Xinyu Wang, Yang Tian, Shuang Song

**Affiliations:** ^1^ College of Food Science and Technology, Yunnan Agricultural University, Kunming, China; ^2^ Yunnan Key Laboratory of Precision Nutrition and Personalized Food Manufacturing, Yunnan Agricultural University, Kunming, China; ^3^ Engineering Research Center of Development and Utilization of Food and Drug Homologous Resources, Ministry of Education, Yunnan Agricultural University, Kunming, China

**Keywords:** *Moringa oleifera* leaves protein, immunosuppression, Jurkat cells, cell proliferation, MAPK/AKT signaling pathway, autophagy

## Abstract

Acute lymphoblastic leukemia (T-ALL) is one of the most common hematologic malignancies in children worldwide. Despite advances in chemotherapy in recent years, more than 50% of adult T-ALL patients still experience treatment resistance and relapse/refractory disease. *Moringa oleifera*, a new food resource in China, has high nutritional value and various pharmacological activities. *Moringa oleifera* protein is one of the main active ingredients in *Moringa oleifera* leaves, and some studies have shown that *Moringa oleifera* protein has antioxidant, anti-inflammatory, antibacterial, anticancer, and other bioactivities; however, the anti-acute lymphoblastic leukemia (T-ALL) activity of the *Moringa oleifera* protein and its mechanism of action are still unclear. In this study, we used *Moringa oleifera* leaves protein as the experimental material and used the MTT assay, flow cytometry, and Western blot techniques to study the effects of *Moringa oleifera* leaves protein on Jurkat cell growth, apoptosis, and the cell cycle *in vitro* and the underlying molecular mechanism. *Moringa oleifera* leaves protein inhibited Jurkat cell proliferation; induced apoptosis and cycle arrest; increased autophagy; and inhibited Jurkat cell proliferation by regulating the MAPK/AKT pathway. The results of this study provide new ideas and a theoretical basis for the development of *Moringa oleifera* leaves protein for the prevention and treatment of leukemia.

## Graphical Abstract

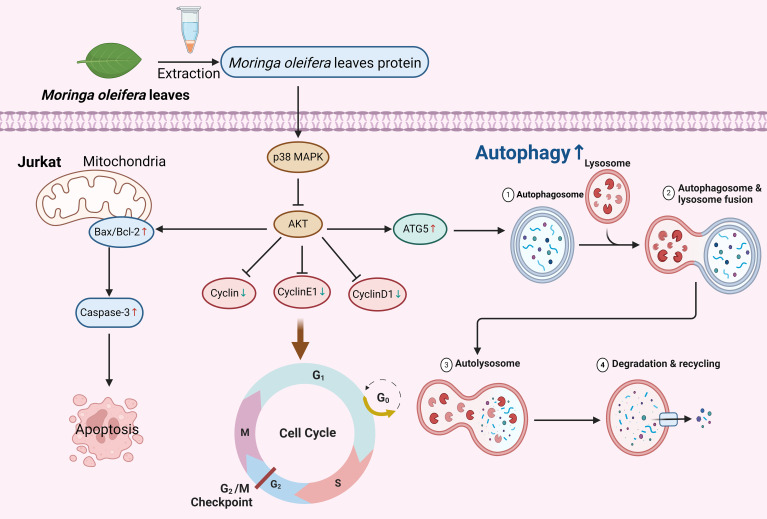

## 1 Introduction

*Moringa oleifera (M. oleifera)*, also known as oil chili wood or drumstick tree, is a perennial tropical deciduous tree of the genus *Moringa* in the family Moraceae (1). *Moringa oleifera* leaves, as one of the main edible parts, have high nutritional value and are rich in protein, fiber, vitamins, a variety of minerals and other nutrients, as well as low-fat, low-cholesterol nutrients, which contain few antinutrients and can effectively resist malnutrition (2). Studies have shown that the protein content of *M. oleifera*, is 20.49%, and *M. oleifera* contains 11 kinds of essential amino acids that the human body cannot synthesize on its own or whose rate of synthesis cannot satisfy its own needs (3). *M. oleifera* leaves are widely used as basic food in some countries and were approved by the Ministry of Health of China as a new food resource in 2012, while *M. oleifera* is also listed as a medicinal food resource in China (4, 5).

Acute lymphoblastic leukemia (ALL) is a highly malignant hematological disease caused by B-cell (B-ALL) or T-cell (T-ALL) cells, and T-ALL is a common hematological malignancy caused mainly by the malignant clonal proliferative transformation of T cells (6, 7). The incidence of leukemia is increasing annually, and chemotherapy-based combination therapy is still the main means of treatment for leukemia (8, 9); however, chemotherapeutic drugs have severe toxic side effects, especially the inhibitory effect on bone marrow hematopoiesis, which often leads to the interruption of chemotherapy, and primary or secondary resistance often leads to chemotherapy failure (9, 10). Therefore, the search for highly effective and low-toxicity antileukemia drugs is one of the main research components of current treatments. As an emerging medicinal plant, all parts of *M. oleifera* have high medicinal value (5). It has received widespread attention because of the multiple active ingredients it contains and its low toxicity. Research on moringa protein has focused mainly on water purification (11–13), antioxidant activity (14), and antibacterial and anti-inflammatory activity (15, 16). Sreelath et al. (17) reported that aqueous extracts of *M. oleifera* leaves have certain inhibitory effects on human tumor cells and induce tumor cell death. Tiloke C et al. (18) reported that aqueous extracts of moringa leaves were able to reduce the value added to A549 human tumor cells. Al-Asmari et al. (19) reported that *M. oleifera* and bark alcohol extracts significantly decreased the survival, colony formation, and migration rates of human colon cancer HCT-8 cells, thereby inducing apoptosis. The survival, colony formation and migration of human colon cancer HCT-8 cells were significantly reduced by the extracts of *M. oleifera* leaves and bark alcohol. These studies suggest that *M. oleifera* has anticancer activity; however, the anti-acute lymphoblastic leukemia (T-ALL) activity and mechanism of action of *M. oleifera* proteins are still unclear.

MAPK/AKT, as a classical cellular signal transduction pathway, plays an important role in cell proliferation and differentiation and apoptosis. Three parallel MAPK signaling pathways, p38MAPK, JNK/SAPK and ERK signaling pathways, have been identified in cells, which play important roles in cell growth, differentiation, inflammatory response and apoptosis and other stress responses (10). T-ALL is characterized by dysregulation of critical signaling pathways, including NOTCH1, PI3K/AKT/mTOR, and JAK/STAT, which play pivotal roles in T-cell development and leukemogenesis. For instance, NOTCH1 mutations are observed in over 50% of T-ALL cases, leading to constitutive activation of the pathway and promoting cell proliferation and survival. Similarly, the PI3K/AKT/mTOR pathway is frequently hyperactivated in T-ALL, contributing to chemotherapy resistance and disease progression. Targeting these pathways has emerged as a promising strategy for T-ALL treatment.

*M. oleifera* has garnered attention for its diverse bioactive compounds, including proteins, flavonoids, and phenolic acids, which exhibit antioxidant, anti-inflammatory, and anticancer properties. Recent studies have demonstrated the potential of *M. oleifera* extracts to inhibit tumor cell proliferation and induce apoptosis in various cancer models, suggesting its promise as a source of anticancer agents. However, the anti-T-ALL activity of Moringa oleifera proteins and their underlying mechanisms remain poorly understood. This study aims to investigate the inhibitory effects of *M. oleifera* leaves protein on T-ALL cells and elucidate its molecular mechanisms, providing a foundation for the development of novel leukemia therapeutics.

M. n this work, *M. oleifera* protein was used as the experimental material, and the T-ALL cell line Jurkat was used as the research object to explore the inhibitory effect of *M. oleifera* leaves protein on the growth of Jurka cells and to elucidate the mechanism of its action for the development of leukemia therapeutic drugs to provide a theoretical basis and experimental basis and a basis for the development of moringa resources.

## 2 Materials and methods

### 2.1 Materials and chemicals

Fresh *M. oleifera* leaves were obtained from Tianyou Technology Co. of Dehong State. *M. oleifera* leaves protein was extracted according to previous methods (20). Human T lymphoblastic leukemia cells (Jurkat) were obtained from the Cell Bank of the Chinese Academy of Sciences. Thiazole blue (MTT) was purchased from Sigma (298-93-1, USA). Phosphate buffer solution (PBS, P1010 ), penicillin-streptomycin solution, high-efficiency cell/tissue lysis solution (RIPA lysis buffer, R0010), dimethyl sulfoxide (DMSO, D8371), penicillin-double antibody, sheep anti-rabbit IgG-HRP (SE134), sheep anti-mouse IgG-HRP (SE131), and a two-color prestained marker were purchased from Beijing Solaibao Technology Co. (Beijing, China). A BCA protein quantification kit was purchased from Beyotime (P0009, Beijing, China). An Annexin V-FITC/PI Apoptosis Detection Kit (40302ES50)and a cell cycle assay kit (40301ES60) from Yeasen Biotechnology Co., Ltd. (Shanghai, China) were used. Bax (AF0120), Bcl-2 (AF6139), cleaved-CasPase-3 (AF7022), CyclinE1 (AF0144), and ATG-5 (DF6010) antibodies were purchased from Affinity Biosciences (OH, USA). Cyclin D1 (WL01435a), ERK1/2 (WL01864), p-ERK1/2 (WLP1512), p-AKT (WLP001a), AKT (WL0003b), p-P38 (WLP1576), and p38 (WL00764) antibodies were obtained from Wanleibio (Shenzhen, China). Anti-β-Tubulin (M20005) was purchased from Abmart Medical Technology (Shanghai, China). All other reagents used in this work were of analytical grade.

### 2.2 Determination of cell viability via the MTT assay

Jurkat cells in the logarithmic growth phase were inoculated into 96-well culture plates at 1×10^5^/well, and a total of 200 μL of *M. oleifera* leaves proteins (0, 30, 60, 90, 120, or 150 μg/mL) and denatured proteins at 150 μg/mL were added under aseptic conditions. Six parallels were set up in each group. After incubation for 24, 48 and 72 h, 20 μL of 5 mg/mL MTT was added, and the cells were incubated at 37°C for 4 h. After centrifugation at 2800 rpm for 10 min, the supernatant was removed, 100 μL of DMSO was added, and the absorbance was measured by shaking the plate with an enzyme counter for 10 min at 570 nm. The survival rate of the cells in each group was calculated as follows: cell survival rate (%) = (absorbance value of drug group - absorbance value of DMSO)/(absorbance value of control group - absorbance value of DMSO).

### 2.3 Apoptosis rate assay

- Cells in the logarithmic growth phase were inoculated in 6-well plates at 2×10^6^ cells per well, and *M. oleifera* leaves protein (0, 30, 60, or 90 μg/mL) was added without using concentration under aseptic operation, with a cell volume of 2 mL per well, and cultured in a constant incubator with 5% carbon dioxide at 37°C.
- After 24 h of culture, the cells were removed, centrifuged at 300 × g for 5 min, the medium was discarded, 2 mL of precooled PBS was added, the mixture was washed twice at 300 × g each time, the mixture was centrifuged for 5 min, the PBS was aspirated and discarded, and 100 μL of 1× binding buffer was added to resuspend the cells. Five microliters of Annexin V-FITC and 10 μL of PI staining solution were added, and the mixture was mixed gently. The mixture was protected from light and allowed to react for 15 min at room temperature.
- A total of 400 μL of 1× binding buffer was added, the mixture was mixed well, the mixture was filtered with 200 mesh gauze, the mixture was placed on ice, and the sample was detected by flow cytometry within 1 h (21).

### 2.4 Cell cycle assay

- Cells were cultured and treated as described in section 2.3 a.
- After 24 h of incubation, the cells were removed, 1000 rPm was added, the mixture was centrifuged for 5 min, the cells were precipitated, and the medium was discarded. One milliliter of precooled PBS was added to resuspend the cells, which were transferred to a 1 mL centrifuge tube. The tube was subsequently centrifuged again to precipitate the cells, the supernatant was discarded, and 300 μL of precooled PBS was added again to resuspend the cells.
- A total of 700 μL of precooled 70% ethanol was added to the cell suspension drop by drop while vortexing at low speed, mixed well and fixed at 4°C for 24 h. The mixture was centrifuged to precipitate the cells the next day, the supernatant was discarded, 2 mL of precooled PBS was added to resuspend the cells, the mixture was centrifuged to precipitate the cells again, and the supernatant was carefully aspirated to avoid aspirating the cells. (The bottom of the centrifuge tube was gently flicked to properly disperse the cells and avoid clumping.) Next, 400 μL of propidium iodide staining solution (propidium iodide staining solution: 400 μL of staining buffer for one sample, 15 μL of propidium iodide staining solution (25×), and 4 μL of RNase A) were added, the cellular precipitates were resuspended slowly and sufficiently, the cells were incubated at 37°C away from light for 30 min, placed on ice, and detected by flow cytometry, and the data were analyzed with FlowJo software.

### 2.5 Immunoblot analysis

Jurkat cells in the logarithmic growth phase were inoculated in small dishes at 3×10^6^ cells per well, and *M. oleifera* leaves protein (0, 30, 60, or 90 μg/mL) were added at a volume of 4 mL per dish without concentration under aseptic conditions. The cells were subsequently cultured in an incubator with 5% carbon dioxide at a constant temperature of 37°C. The cells were then incubated for 24 h at 1500 rPm and centrifuged for 3 min. After 24 h of incubation, the cells were removed and centrifuged at 1500 rPm for 3 min, and the medium was discarded. The cells were washed twice with precooled PBS. Proteins were extracted from Jurkat cells via the prepared RIPA lysate, thawed on ice, mixed, and centrifuged transiently; RIPA lysates containing protease inhibitors were prepared, lysed from adherent cells, separated, and transferred to PVDF membranes. The membranes were blocked with blocking solution (5% skim milk) at room temperature for 1 h. The membranes were incubated overnight at 4°C with primary antibodies against Bax (1:2000), Bcl-2 (1:1000), cleaved CasPase-3 (1:1000), CyclinE1 (1:1500), ATG-5 (1:1000), CyclinD1 (1:1200), ERK1/2 (1:1000), p-ERK1/2 (1:1000), p-AKT (1:1000), AKT (1:1000), p-P38 (1:1000), p38 (1:1000), and β-Tubulin (1:5000). The membranes were then washed with TBST, incubated with HRP-conjugated secondary antibodies (1:5000) at room temperature for 2 h, and washed again. The results were analyzed via an enhanced chemiluminescence (ECL) protein blotting detection system and ImageJ software (V1.8.0).

### 2.6 Statistical analysis

All the data in this study are presented as the means from three or more independent experiments mean ± standard error of the mean (SEM). The data were analyzed via GraphPad Prism software (7.0.0), and one-way analysis of variance (ANOVA) was used for comparisons among groups. * *P* < 0.05, *P* < 0.01, *P* < 0.001, *P* < 0.0001 indicates that there is a statistically significant difference.

## 3 Results

### 3.1 Effects of *M. oleifera* leaves protein on the survival of different cancer cells

**Figure 1:**
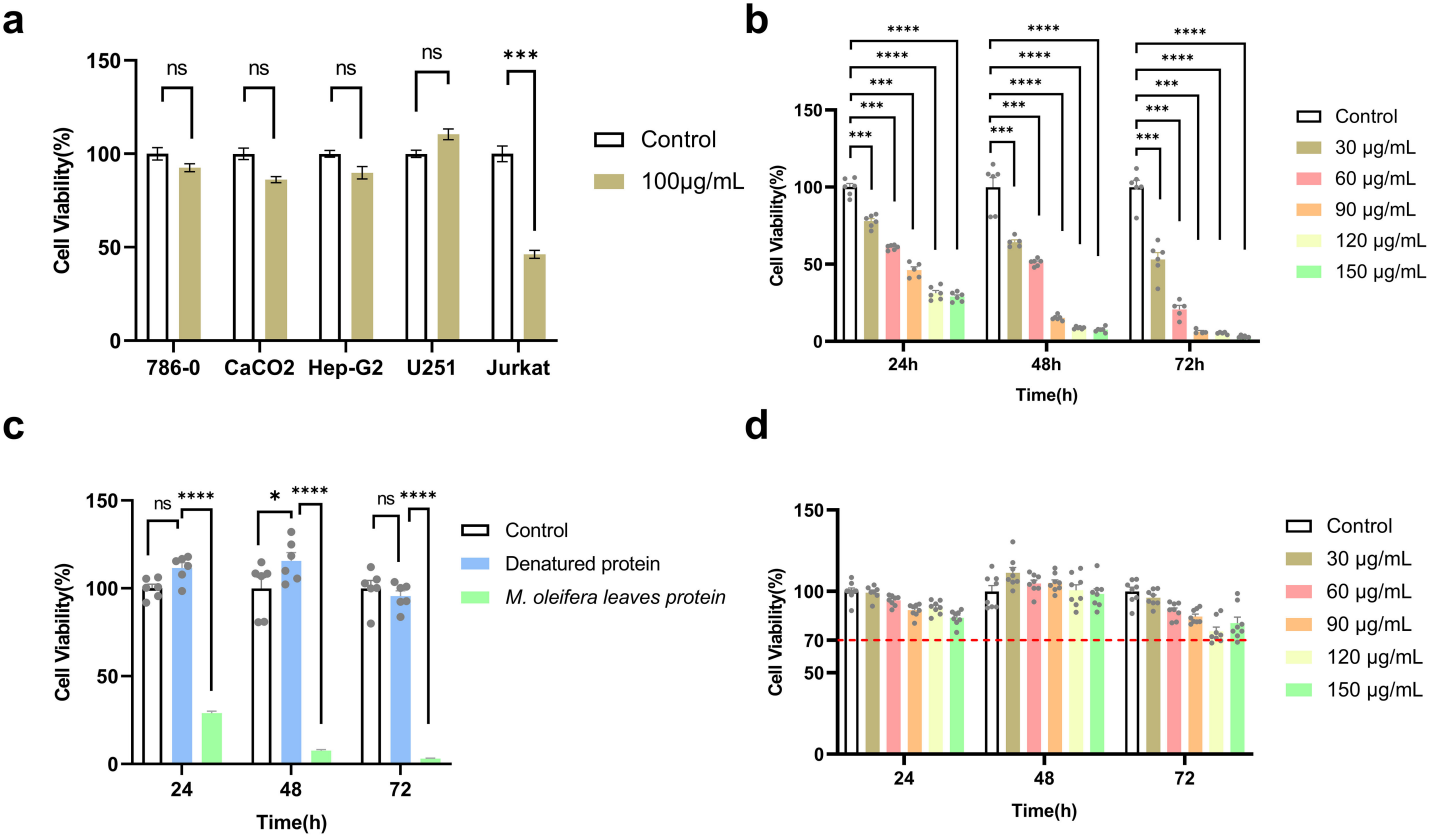
*M. oleifera* leaves protein inhibited the proliferation of Jurkat cells. **(a)** Effect of *M. oleifera* leaves protein on five cancer cell lines; **(b)** effect of *M. oleifera* leaves protein on the human T-lymphoblastic leukemia cell line Jurkat; **(c)** effect of denatured *M. oleifera* leaves protein on the viability of Jurkat cells; **(d)** *M. oleifera* leaves protein on the viability of GES-1 cells. Effect of *M. oleifera* leaves protein on Jurkat and GES-1 cells were treated with 30, 60, 90, 120, or 150 μg/mL *M. oleifera* leaves protein for 24, 48 or 72 h. Values represent the mean ± SEM of the mean; ****P < 0.0001; ***P < 0.001; ns indicates no significant difference. For each group, the data were from 3 separate experiments and 6 parallel samples (n =6).

A previous study revealed that after treating five types of cancer cells (786-0, CaCO2, HePG2, U251, and Jurkat) with 100 μg/mL *M. oleifera* leaves protein for 24 h, the growth of U251 and Jurkat cells was significantly inhibited by moringa proteins, with the strongest inhibitory effect on the growth of Jurkat cells, which was followed by the experiments with Jurkat cells as test subjects (**Figure 1a**).

### 3.2 *M. oleifera* leaves proteins inhibit the proliferation of Jurkat cells

After 24 h of treatment of Jurkat cells with *M. oleifera* leaves protein (30, 60, 90, 120, or 150 μg/mL) at different concentrations under aseptic conditions, the cell survival rate was detected by MTT, and compared with that of the blank control group (0 μg/mL), the cell survival rate decreased from 100% to 77.93% (*P* < 0.001), 60.93% (*P* < 0.001), 46.21% (*P* < 0.001), 31.23% (*P* < 0.0001), and 28.90% (*P* < 0.0001), respectively. The IC50 at 24 h was 77.01 μg/mL. After treatment at the same concentration for 48 h, the cell survival rates decreased to 58.72% (*P* < 0.001), 46.69% (*P* < 0.001), 13.88% (*P* < 0.0001), 7.94% (*P* < 0.0001), and 6.88% (*P* < 0.0001), with an IC50 of 41.26 μg/mL. After 72 h of treatment at the same concentration, the cell survival rates decreased to 53.11% (*P* < 0.001), 20.75% (*P* < 0.001), 6.29% (*P* < 0.001), 5.31% (*P* < 0.0001), and 3.03% (*P* < 0.0001). IC50 = 31.87 μg/mL. The experimental results revealed that *M. oleifera* leaves protein decreased Jurkat cell survival in a time- and dose-dependent manner (**Figure 1b**).

After 24 h, Jurkat cells were subjected to different concentrations of *M. oleifera* leaves protein (0, 30, 60, or 90 μg/mL) for processing, and the morphology of the cells was observed under an inverted microscope. As shown in **Supplementary Figure S1**, compared with that in the blank group, the number of spicy konoha proteins in Jurkat cells markedly changed, and the number of apoptotic bodies increased. In the blank group, the cell bodies were round, similar to those of the fish eggs, and the cytoplasm was uniform, transparent, and refractive (22). They grew in suspension, similar to grape clusters, and aggregated into large colonies. Under high-magnification microscopy, the cell bodies were round, and the nuclei were large and round. In the treatment group, with increasing drug concentration, colony formation and the number of cells significantly decreased, the number of single suspended cells significantly increased, the cell size significantly differed, the cell body shrank, the number of cell particles increased, the refraction weakened, the overall outline of the cell was unclear, and nuclear contraction, cytoplasmic reduction and cytoplasmic vacuoles were observed at high magnification (23). The results showed that *M. oleifera* leaves protein could change the morphology of Jurkat cells.

Because the proposed *M. oleifera* leaves protein contains pigments and other substances, to clarify whether the reduction in Jurkat cell viability was caused by *M. oleifera* leaves protein or other substances, the denatured moringa leaf protein (121°C, treated for 30 min) was used to treat the Jurkat cells for 24 h, 48 h and 72 h. Compared with that of the blank group (0 μg/mL), the cell survival rates were 100%, 111.51%, 115.51%, and 95.56%, respectively (**Figure 1c**), and the cell viability was above 95% in all three periods, indicating that the main substance causing the decrease in the viability of Jurkat cells was *M. oleifera* leaves protein.

To determine whether *M. oleifera* has toxic effects on normal cells, human gastric mucosa cells (GES-1) were treated with different concentrations (0, 30, 60, or 90 μg/mL) of *M. oleifera* leaves protein, and the survival rate of the cells was measured via the MTT method. As shown in **Figure 1d**, compared with those of the blank control group (0 μg/mL), the cell survival rates were 100%, 99.23%, 94.39%, 88.42%, 90.15%, 83.85%, and 89.36%, respectively. After treatment with the same concentration, the cell survival rates at 48 h were 100%, 111.40%, 104.23%, 105.18%, 100.66% and 115.76%, respectively. At 72 h, treatment with 120 μg/ml and 150 μg/ml of Moringa oleifera leaf protein resulted in significant reductions in Jurkat cell viability to approximately 60% (*P* < 0.01) and 70% (*P* < 0.05), respectively, compared to the control group.

### 3.3 *M. oleifera* leaves protein induced Jurkat cell apoptosis

**Figure 2:**
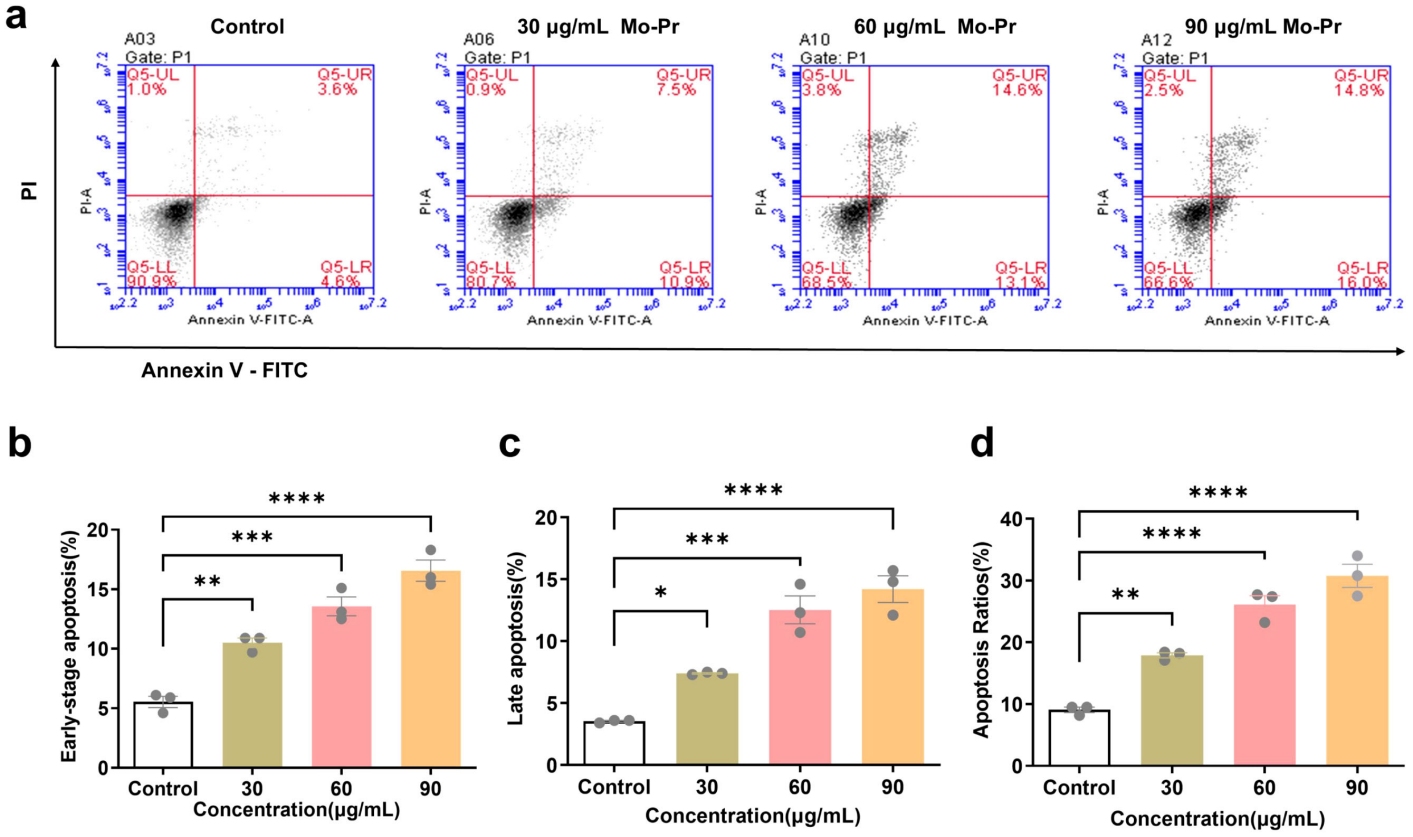
Effects of *M. oleifera* leaves protein on the apoptosis of Jurkat cells. **(a)** Effect of *M. oleifera* leaves protein on Jurkat cell apoptosis. **(b)** Early apoptosis. **(c)** Late apoptosis. **(d)** Total number of apoptotic cells. Compared with the blank control group (0 μg/mL), the difference between groups is expressed as *P < 0.05, **P < 0.01, ***P < 0.001, ****P < 0.0001.

After the Jurkat cells were treated with different concentrations of *M. oleifera* leaves protein (0, 30, 60, or 90 μg/mL) for 24 h, the effect of *M. oleifera* leaves protein on the apoptosis of Jurkat cells was detected via flow cytometry. As shown in **Figure 2a**, the apoptosis rate increased with increasing drug concentration, and the early apoptosis rate (**Figure 2b**) increased from 5.33% to 10.5% (*P* < 0.01), 13.57% (*P* < 0.001), and 18.3% (*P* < 0.0001), respectively. The late apoptosis rate (**Figure 2c**) increased from 3.53% to 7.4% (*P* < 0.05), 12.53% (*P* < 0.001), and 14.2% (*P* < 0.0001), respectively. The total apoptosis rate (**Figure 2d**) increased from 9.07% to 17.9% (*P* < 0.01), 26.1% (*P* < 0.0001), and 30.77% (*P* < 0.0001), respectively.

### 3.4 Effects of *M. oleifera* leaves protein on the Jurkat cell cycle

**Figure 3:**
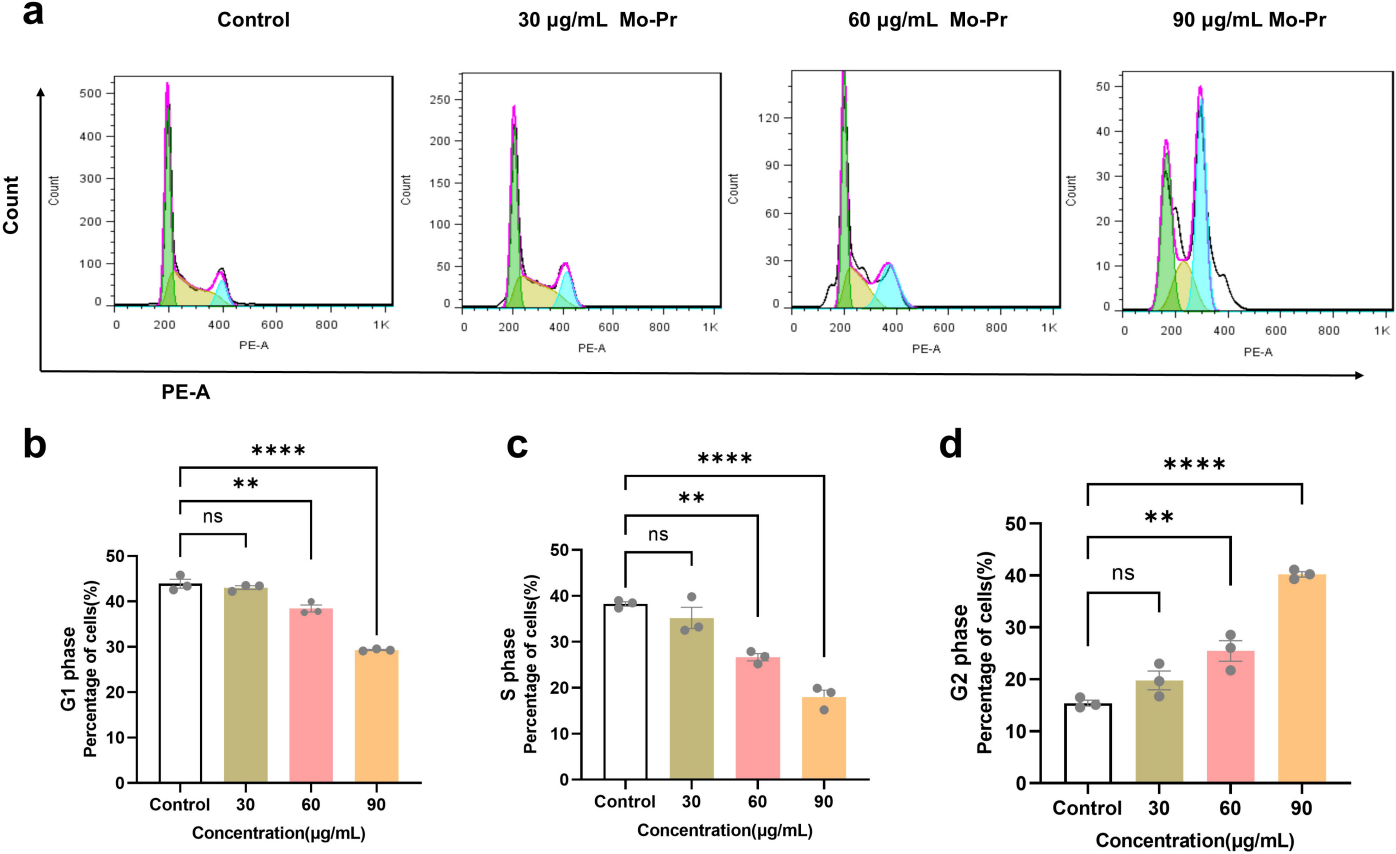
Effects of *M. oleifera* leaves protein on the Jurkat cell cycle. **(a)** Effects of *M. oleifera* leaves protein on the Jurkat cell cycle. **(b)** Effects of *M. oleifera* leaves protein on the G1 phase of Jurkat cells. **(c)** Effects of *M. oleifera* leaves protein on the S phase of Jurkat cells. **(d)** Effects of *M. oleifera* leaves protein on the G2 phase of Jurkat cells. Compared with the blank control group (0 μg/mL), the differences between the groups are expressed as **P < 0.01 and ****P < 0.0001. ns indicates no significant difference.

*The* cell cycle refers to the whole process through which a cell progresses from the completion of one division to the end of the next division, which is divided into two stages: the interphase and division phases. The interphase is divided into three stages, namely, the early phase of DNA synthesis (G1 phase), the DNA synthesis phase (S phase) and the late phase of DNA synthesis (G2 phase). The G1 phase (first gap) is the period from mitosis to DNA replication, also known as the prosynthetic phase, in which RNA and ribosomes are synthesized. This stage is characterized by active substance metabolism, rapid synthesis of RNA and protein, and a significant increase in cell volume. The main purpose of this phase is to prepare the material and energy for the next phase of DNA replication, phase S. S phase (synthesis) is the DNA synthesis phase, in addition to the synthesis of DNA and the synthesis of histones. The enzymes needed for DNA replication are synthesized during this period. The G2 phase (second gap) is the late stage of DNA synthesis, which is the preparatory stage for mitosis. During this period, DNA synthesis stops, and many RNA and proteins, including tubulin and maturation-promoting factors, are synthesized. As shown in **Figure 3**, *M. oleifera* leaves protein induced the apoptosis of Jurkat cells, and the concentration of *M. oleifera* leaves protein was 0, 30, 60, or 90 μg/mL after the Jurkat cells were treated for 24 h. The percentage of G1-phase cells increased from 43.92% to 43.05%, 38.46% (*P* < 0.01), and 29.31% (*P* < 0.001), and the percentage of S-phase cells increased from 38.27% to 35.20%, 26.64% (*P* < 0.01), and 18.02% (*P* < 0.001), respectively. The number of cells in the G2 phase increased from 15.41% to 19.79%, 25.46% (*P* < 0.01), and 40.21% (*P* < 0.00), the number of cells in the G1 phase and S phase decreased significantly, and the number of cells in the G2 phase increased significantly, indicating that *M. oleifera* leaves protein blocked the proliferation of Jurkat cells in the G2/M phase.

### 3.5 Effects of *M. oleifera* leaves protein on the expression of apoptotic proteins, cyclins and autophagy proteins in Jurkat cells

Apoptosis, also known as programmed cell death, involves several signaling pathways and genes involved in the process of apoptosis, among which the Bcl-2 protein family and the Caspase series of genes are known to be the most classical cell signaling pathways (24). There are antiapoptotic members of the Bcl-2 protein family, such as Bcl-2 and MCL-1. Bcl-2 is the most representative apoptosis-inhibiting gene and is abundantly expressed in malignant tumors such as gastric cancer and leukemia. It plays an important regulatory role in tumor cells (25). An increase in Bcl-2 then prevents the release of cytochrome C from the mitochondria to the cytoplasm, thereby inhibiting apoptosis (26). The release of apoptosis-inducing factors can deregulate the inhibition of Caspase-9 and activate Caspase-9 and eventually Caspase-3, thus inducing apoptosis (27). Caspase-3 is an important member of the apoptosis family of caspases and is essentially a nucleic acid endonuclease that functions in the execution of apoptosis (28). Studies have shown that CyclinD1 is a central factor in the endogenous regulation of the cell cycle (29). CyclinD1 drives the transformation of cells from the G1 phase to the S phase and initiates the progression of cell division and the proliferation cycle (30). Atg5 is an autophagy hallmark protein, and its level often reflects the level of autophagosome formation.

**Figure 4:**
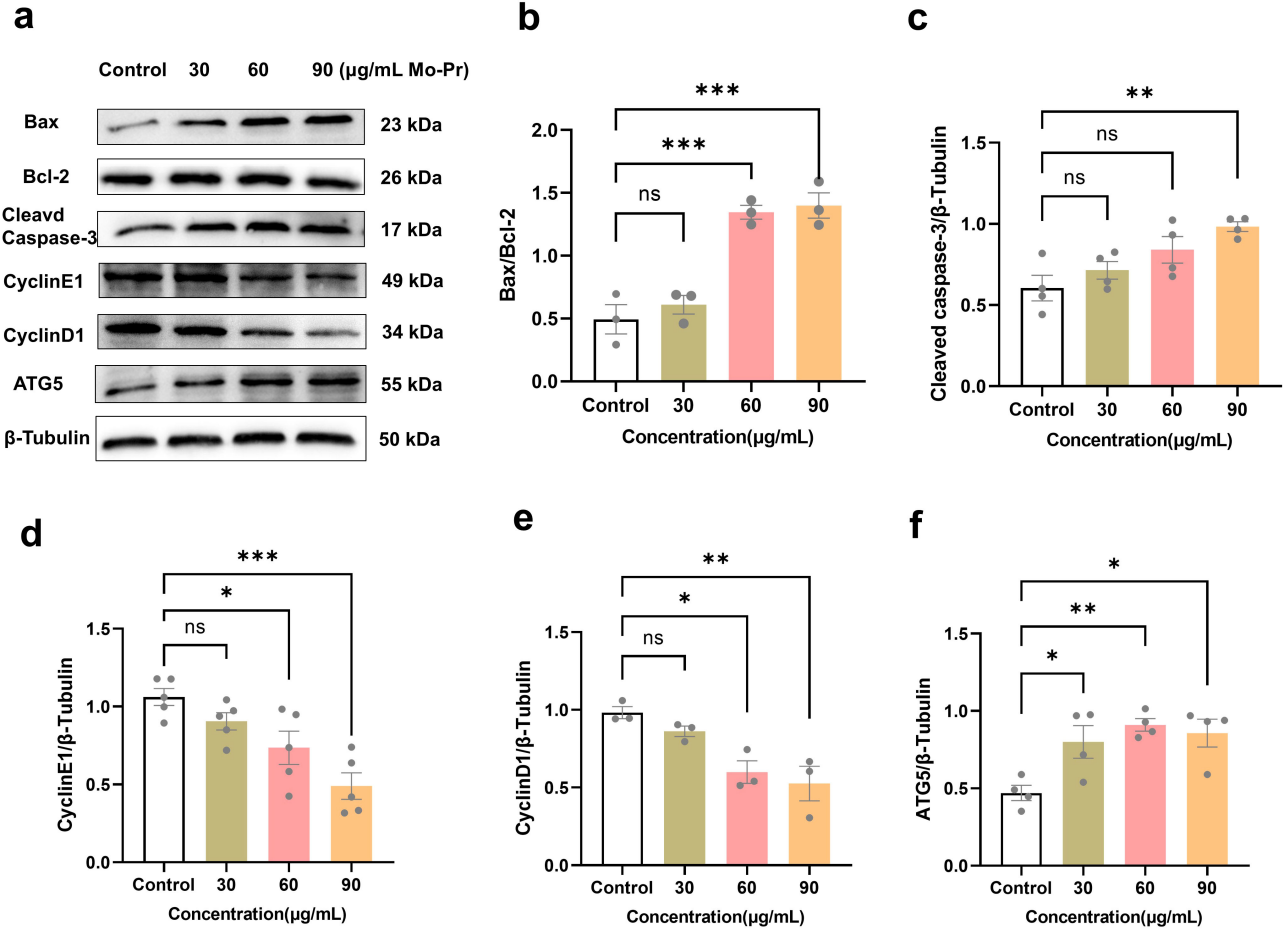
Effects of *M. oleifera* leaves protein on Jurkat cell apoptosis and cyclin and autophagy protein expression. **(a)** Effects of *M. oleifera* leaves proteins on the expression of apoptosis, cyclin and autophagy proteins in Jurkat cells. **(b)** Bax/Bcl-2, **(c)** cleaved CasPase-3, **(d)** CyclinE1, **(e)** CyclinD1 and **(f)** ATG5 statistical analysis of protein expression. Compared with the blank control group (0 μg/mL), the differences between the groups are represented by *P<0.05, **P<0.01, and ***P<0.001. ns indicates no significant difference.

Jurkat cells were treated with different concentrations of *M. oleifera* leaves protein (0, 30, 60, or 90 μg/mL) for 24 h. Western blotting was used to detect changes in the expression of the apoptotic proteins Bax/Bcl-2, cleaved CasPase-3, the cyclins CyclinE1 and CyclinD1, and the apoptotic protein ATG-5 in cells. As shown in **Figure 4**, compared with the blank control (0 μg/mL), the concentration (30, 60, or 90 μg/mL) of *M. oleifera* leaves protein increased the protein expression of the apoptotic proteins Bax/Bcl-2 by 0.23-fold, 1.72-fold (*P* < 0.001) and 1.83-fold (*P* < 0.001), cleaved CasPase-3 by 0.18-fold, 0.33-fold and 0.45-fold (*P* < 0.01), and cyclin E1 expression decreased 0.23-fold, 0.49-fold (*P* < 0.05) and 0.69-fold (*P* < 0.001), respectively. In addition, cyclin D1 expression decreased 0.12-fold, 0.39-fold (*P* < 0.05) and 0.46-fold (*P* < 0.01). The protein expression of the autophagy protein ATG5 increased 0.46-fold, 0.78-fold (*P* < 0.05) and 0.86-fold (*P* < 0.05).

### 3.6 *M. oleifera* leaves protein affects Jurkat cell growth

Previous experimental results showed that *M. oleifera* leaves protein could inhibit the proliferation of Jurkat cells and induce cell apoptosis and cell cycle arrest. Therefore, the potential mechanism of action was further explored to further determine the role of the AKT, ERK and p38 signaling pathways in Jurkat cells induced by *M. oleifera* leaves protein. *M. oleifera* leaves protein was used to treat Jurkat cells for 24 h to detect changes in the protein expression levels of the MAPK/AKT signaling pathway in Jurkat cells.

The MAPK/AKT pathway plays a key role in promoting cell survival (31). The MAPK pathway can be activated by various cell stresses and growth factors and is a major signal transduction molecule for apoptosis (31). MAPK signaling pathways, including erk, p38mapk (P38) and jnk, have been identified as chemical targets that sensitize cancer cells to apoptosis (32).

**Figure 5:**
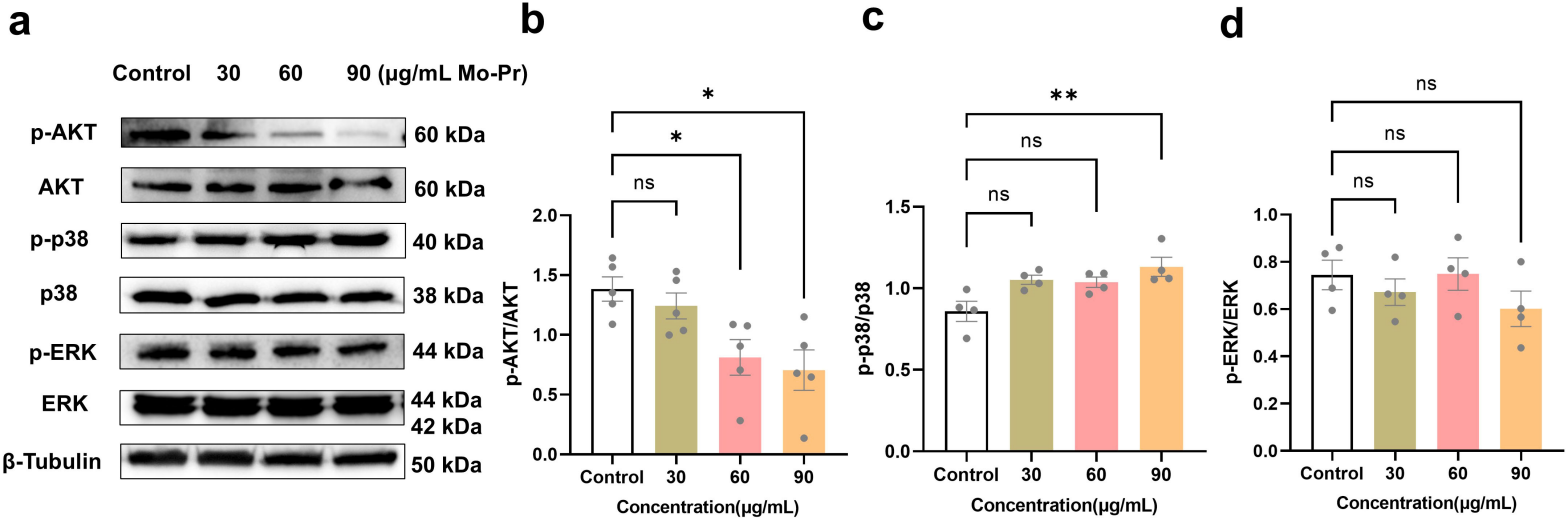
*M. oleifera* leaves protein regulates the growth mechanism of Jurkat cells. **(a)** Effects of *M. oleifera* leaves protein on the expression of pathway-related proteins. **(b)** -AKT/AKT, **(c)** p-p38/p38, and **(d)** p-ERK/ERK ratios were statistically analyzed for protein expression. Compared with the blank control group (0 μg/mL), the differences between the groups are represented by *P<0.05 and **P<0.01. ns indicates no significant difference.

As shown in **Figure 5**, compared with the blank control (0 μg/mL), *M. oleifera* leaves protein decreased the expression of P-Akt protein in Jurkat cells by 0.10-fold, 0.41-fold (*P* < 0.05) and 0.49-fold (*P* < 0.05). The protein expression of P-P38 increased by 0.23, 0.21 and 0.32 times, respectively (*P* < 0.05). The results revealed that moringa leaf protein could significantly reduce the content of p-AKT and increase the content of p-p38, but there was no significant change in the content of p-ERK, indicating that *M. oleifera* leaves protein could significantly regulate the MAPK/AKT signaling pathway and promote the apoptosis of Jurkat cells.

## 4 Discussion

*M. oleifera* leaves protein is widely recognized as a high-quality plant-based protein, boasting a protein content ranging from 10.74% to 30.29% (33). This remarkable protein profile positions *M. oleifera* leaves protein as one of the most nutritionally valuable plant proteins known to date. Beyond its nutritional benefits, *M. oleifera* leaves protein has garnered significant attention for its potential therapeutic applications, particularly in cancer research. Unlike many other plant proteins, *M. oleifera* leaves protein exhibits unique bioactive properties that may contribute to its anticancer effects. These properties are particularly relevant given the growing interest in plant-derived compounds as safer and more sustainable alternatives to conventional chemotherapeutic agents. The anticancer potential of plant proteins varies significantly depending on their source and composition. For instance, Shan Shuhua et al. (34) demonstrated that corn husk protein selectively targets colon cancer cells while sparing normal cells, highlighting its potential as a safe and effective anticancer agent. Similarly, Fan Handong et al. (35) reported that hemicellulose protein inhibits the growth of the tumor cell line Sarcoma180 in a concentration-dependent manner, with higher concentrations yielding greater inhibition rates. Li Penggao et al. (36) further expanded on this by showing that sweet potato proteins suppress the proliferation of multiple tumor cell lines, including Hep-G2, Bcap-37, and HT-29, in a dose-dependent fashion. These studies collectively underscore the diverse mechanisms through which plant proteins exert their anticancer effects, ranging from direct cytotoxicity to the modulation of cell survival pathways. In this study, we investigated the anticancer potential of *M. oleifera* leaves protein using Jurkat cells, a human T-lymphocyte cell line commonly employed as a model for studying T-cell malignancies. Treatment of Jurkat cells with *M. oleifera* leaves protein at concentrations of 30, 60, 90, 120, or 150 μg/mL for 24, 48, or 72 hours revealed a significant reduction in cell viability, even at the lowest concentration of 30 μg/mL. Furthermore, *M. oleifera* leaves protein treatment markedly inhibited the formation of cell colonies, providing compelling evidence of its ability to suppress Jurkat cell proliferation. These findings align with previous reports on the anticancer properties of plant proteins but also highlight the unique potency of *M. oleifera* leaves protein, which exerts significant effects at relatively low concentrations. To elucidate the mechanisms underlying *M. oleifera* leaves protein’s anticancer effects, we examined its impact on cell cycle progression and apoptotic pathways. Wang Y et al. (37) previously demonstrated that phycocyanin, a pigment-protein complex, induces G1 phase arrest in HeLa cells, thereby preventing DNA replication and cell division. Similarly, Fan Handong et al. reported that hemicellulose proteins block Sarcoma180 cells in the G0/G1 phase, with the proportion of arrested cells increasing in a concentration-dependent manner. In contrast, our study revealed that *M. oleifera* leaves protein treatment induces G2/M phase arrest in Jurkat cells, a distinct mechanism that may enhance its efficacy against rapidly proliferating cancer cells. This phase-specific arrest was accompanied by significant morphological changes indicative of apoptosis, including cell shrinkage and membrane blebbing. At the molecular level, *M. oleifera* leaves protein treatment upregulated the expression of pro-apoptotic proteins Bax and cleaved Caspase-3 while downregulating the anti-apoptotic protein Bcl-2, tipping the balance toward programmed cell death. Additionally, *M. oleifera* leaves protein suppressed the expression of cyclin proteins CyclinE1 and CyclinD1, which are critical regulators of cell cycle progression. Concurrently, *M. oleifera* leaves protein upregulated the expression of ATG5, a key autophagy-related protein, suggesting that autophagy may also contribute to its anticancer effects. These findings collectively demonstrate that *M. oleifera* leaves protein exerts its anticancer activity through a multifaceted approach, involving cell cycle arrest, apoptosis induction, and autophagy activation. The induction of apoptosis in cancer cells is often mediated through the dysregulation of key signaling pathways. Yang Tingfang et al. (38) reported that andrographolide inhibits Jurkat cell growth by downregulating the PI3K/AKT pathway and upregulating the p38MAPK pathway. Similarly, Linjie Zeng et al. (39) found that Ganoderma lucidum lectin activates the p38/JNK and MAPK pathways to induce apoptosis in Jurkat cells. Liu Bin et al. (40) further demonstrated that arsenic trioxide (As2O3) modulates the MAPK/AKT pathway by increasing p-p38 and p-JNK levels while decreasing p-AKT levels in Jurkat cells. In line with these studies, our results revealed that *M. oleifera* leaves protein treatment significantly reduced p-AKT expression and increased p-p38 expression in Jurkat cells, with no significant changes in p-ERK levels (41). These findings suggest that *M. oleifera* leaves protein promotes apoptosis in Jurkat cells by modulating the MAPK/AKT signaling pathway, a mechanism that aligns with the actions of other well-studied anticancer agents. In addition to its direct anticancer effects, *M. oleifera* leaves protein treatment resulted in the suppression of Jurkat cell proliferation, indicating potential immunosuppressive properties. This effect is particularly relevant in the context of T-cell malignancies, where uncontrolled proliferation of T-lymphocytes is a hallmark of disease progression. By inhibiting Jurkat cell growth and inducing apoptosis, *M. oleifera* leaves protein may serve as a promising therapeutic agent for T-cell-related disorders, offering a dual benefit of anticancer and immunomodulatory activity.

In summary, our study demonstrates that *M. oleifera* leaves protein exerts significant anticancer effects on Jurkat cells through multiple mechanisms, including cell cycle arrest, apoptosis induction, and autophagy activation. These effects are mediated, at least in part, by the modulation of the MAPK/AKT signaling pathway. While these findings are promising, further research is needed to validate the efficacy of *M. oleifera* leaves protein *in vivo* and explore its potential applications in combination therapies (42). While this study demonstrates the involvement of the MAPK/AKT signaling pathway in the apoptosis of Jurkat cells, further investigation is needed to elucidate the precise molecular mechanisms by which M. oleifera leaves protein modulates these pathways. For instance, how does the protein interact with upstream regulators of these pathways, and are there additional signaling cascades involved? Also, the exploration of the molecular mechanisms of downstream mTOR signaling (43). Additionally, future studies should investigate the effects of *M. oleifera* leaves protein on other cancer cell lines and its broader implications for cancer treatment and prevention. The current study is limited to *in vitro* experiments. Future research should include *in vivo* studies to evaluate the efficacy, safety, and pharmacokinetics of *M. oleifera* leaves protein in animal models. This would provide critical insights into its potential as a therapeutic agent in a physiological context. Detailed structural analysis of the bioactive components in *M. oleifera* leaves protein could help identify specific peptides or domains responsible for its anticancer activity. This could pave the way for the development of synthetic analogs or peptide-based drugs.

## 5 Conclusions

In this study, we used *M. oleifera leaves* protein as a raw material and Jurkat cells, a T-ALL cell line, as experimental subjects and found that *M. oleifera* leaves protein decreased Jurkat cell survival and colony formation in a time- and dose-dependent manner, induced early apoptosis, and blocked cell proliferation at the G2/M phase. Increased expression levels of the proapoptotic proteins Bax/Bcl-2 and cleaved caspase-3, decreased expression levels of the cyclins CyclinE1 and CyclinD1, and increased expression levels of the autophagy protein ATG5, but denatured proteins had no effect on Jurkat cells. There was no effect on normal human gastric mucosal cells (GES-1) under the same conditions. The *M. oleifera* leaves protein inhibited the expression level of p-AKT and increased the expression level of p-p38, but there was no significant change in the p-ERK protein content, suggesting that *M. oleifera* leaves proteins inhibited the proliferation of Jurkat cells by regulating the MAPK/AKT pathway.

## Data Availability

The datasets presented in this study can be found in online repositories. The names of the repository/repositories and accession number(s) can be found in the article/**Supplementary material**.
