# Supplementary material for: Moringa oleifera leaves protein suppresses T-lymphoblastic leukemogenesis via MAPK/AKT signaling modulation of apoptotic activation and autophagic flux regulation
Source: Front Immunol. 2025 Apr 1;16:1546189. doi: 10.3389/fimmu.2025.1546189 (PMC11996636; doi:10.3389/fimmu.2025.1546189)
Supplement: Supplementary file 1 [file Image1.pdf]

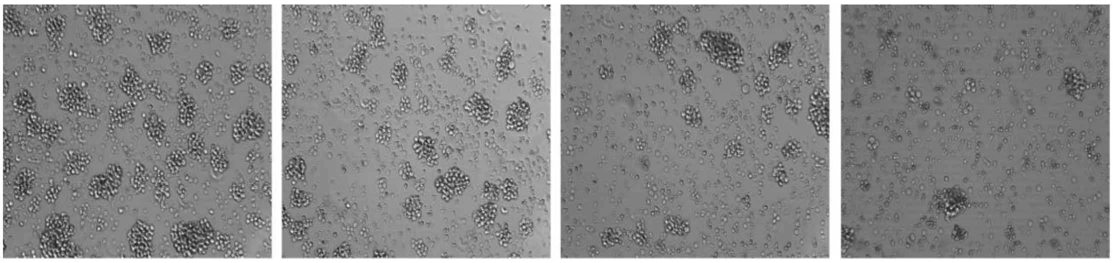

Supplementary Figure S1.The effect of *Moringa oleifera* leaves protein on the morphological changes of Jurkat cells.
